# Neglected Infectious Diseases: Mechanism of Pathogenesis, Diagnosis, and Immune Response

**DOI:** 10.1155/2012/701648

**Published:** 2012-12-27

**Authors:** Eliete Caló Romero, Fabiana Pimenta, Décio Diament

**Affiliations:** ^1^Instituto Adolfo Lutz, Av. Dr. Arnaldo 355, 9o andar, 01246-902 São Paulo, SP, Brazil; ^2^Instituto de Infectologia Emílio RIbas, Av. Dr. Arnaldo, 165 Cerqueira Cesar, 01246-901 São Paulo, SP, Brazil; ^3^Centers for Disease Control and Prevention, Mail Stop C-02, 1600 Clifton Rd N. E., Atlanta, GA 30333, USA

Neglected infectious diseases (NIDs) represent a major global health challenge and comprise a group of endemic diseases which occurs in impoverished regions of Asia, Africa, and America. The World Health Organization (WHO) list of 17 diseases includes Buruli ulcer, Chagas disease, cysticercosis, dengue, dracunculiasis, echinococcosis, fascioliasis, human African trypanosomiasis, leishmaniasis, leprosy, filariasis, onchocerciasis, rabies, schistosomiasis, soil-transmitted helminthiasis, trachoma, and yaws. According to the WHO, neglected infectious diseases affect more than one billion human beings, mainly in tropical areas, in 149 underdeveloped or developing countries [1], imposing great economical burden to its overloaded health systems. Preventive measures and treatments are not widely available in developing countries, despite the low cost of some treatments. Due to globalized trade and travel, some diseases may emerge in developed places or reemerge where they once were controlled [2]. The immediate need is to rise comprehensive control programs based on multidisciplinary approaches that include environmental, therapeutic, diagnoses, and other measures. Also, NIDs may spread into the population in developed countries due to demographic change. Recent advances in biology and medicine have introduced new technologies to study and understand the epidemiology, the reinfection and coinfections, and the mechanisms of the development of resistance to treatments in NIDs. Continuous coordinated efforts are needed in order to achieve these goals, and government and nongovernment [3] programs focused on NIDs are on the agenda.

We invited investigators to contribute original research articles as well as review articles that will stimulate the continuing efforts to understand the NIDs, the development of strategies to treat these diseases, and the evaluation of outcomes. We were particularly interested in articles describing the advances in molecular genetics and molecular diagnostics, new insights into reducing morbidity and mortality, and current concepts in the treatment of NIDs.

In this issue, S. D. Hulme et al. described molecular mechanisms of intracellular murine macrophage survival of pathogenic *Salmonella*. Despite its absence on the WHO list of NIDs, salmonellosis remains an important cause of morbidity and mortality in the developing world, as an agent of acute diarrhea and enteric fever [4]. Two papers, one by A. A. Euzébio et al. and another by N. R. B. Zuim et al., evaluated the pathogenicity of different Brazilian strains of *Schistosoma mansoni* using an experimental murine model, regarding granuloma formation. Differences in pathogenicity could explain distinct clinical evolution of disease in humans, leading to better diagnosis and treatment. D. F. Dávila et al. reviewed physiopathogenic aspects of cardiac involvement in Chagas disease, stressing current theories of myocardial dysfunction. Understanding these issues implies diverse treatments. A. Singh and V. K. Kashyap described a PCR method for *Mycobacterium tuberculosis* detection, using triple DNA targets, resulting in better performance in relation to classical methods of smear and culture. More efficient diagnosis could lead to prompt treatment, avoiding complications of tuberculosis. V. J. Castillo-Morales et al. studied Mexican domestic cat infection with *Toxoplasma gondii* to determine prevalence and risk factors for infection, using molecular and serological methods. A. R. Nimir presented a comprehensive review of ophthalmologic parasitosis, covering protozoan, helmintic, and ectoparasitic etiologies. The eyes are an important source of symptoms and signs and should be always examined in order to promote precocious diagnosis of parasitic diseases. E. Guzman-Marin et al. studied the influence of *Triatoma dimidiata* in modulating the virulence of *Trypanosoma cruzi*. R. M. Bhat and C. Prakash reviewed genetic determinants of host response, clinical aspects, and transmission and immunology of leprosy, which continues to strike poor populations in developing countries, demanding great efforts from the health systems to control it. O. H. Montes et al. analyzed kinetoplast DNA from isolates of *Leishmania mexicana* in order to determine whether a particular minicircle class is exclusive of one strain or if the differences in clinical manifestation are related to any particular minicircles classes. P. Bhargava and R. Singh reviewed tools for leishmaniasis diagnosis, including new molecular and serological methods, and antileishmania drug development, like miltefosine, new amphotericin lipid formulations, new 8-aminoquinolines, and new potential drug targets in parasite metabolic pathways. H. Honarmand made a review of Q fever, a zoonosis that affects several animals and humans.

In closing this introduction to the special issue, we would like to express our gratitude to the contribution of all the authors and reviewers. We sincerely hope that this special issue will stimulate further investigation of neglected infectious diseases.

*Eliete Caló Romero* *Eliete Caló Romero* *Fabiana Pimenta* *Fabiana Pimenta* *Décio Diament* *Décio Diament*
